# Ginger-derived exosome-like nanoparticles: a representative of plant-based natural nanostructured drug delivery system

**DOI:** 10.3389/fbioe.2025.1569889

**Published:** 2025-06-04

**Authors:** Houhe Liu, Yi Deng, Jiayan Li, Wen Lin, Chongzhi Liu, Xiaofei Yang, Zunzhen Zhou, Yuan Jiang

**Affiliations:** ^1^ College of Medicine, Linyi University, Linyi, Shandong, China; ^2^ Clinical Medical College and the First Affiliated Hospital of Chengdu Medical College, Chengdu, Sichuan, China

**Keywords:** plant-derived exosome-like nanoparticles, ginger, ginger-derived exosome-like nanoparticles, drug delivery system, intestinal diseases

## Abstract

In recent years, the research on plant-derived exosome-like nanoparticles (PELNs) has attracted increasing attention. Among these, ginger-derived exosome-like nanoparticles (GELNs) stand out due to their specific pharmacological activity and their role as reliable carriers for delivering both hydrophilic and hydrophobic drugs, as well as small RNAs, making them a noteworthy representative of plant-based natural nanostructured drug delivery systems (DDS). In this review, we first introduce the characteristics and engineering methods of GELN-based DDS to brush up on our current understanding and then focus on research progress to summarize their therapeutic application scope and challenges.

## 1 Introduction

Ginger (*Zingiber officinale Roscoe*) is a traditional herbal plant abundant in bioactive compounds, including phenolic and terpene (Zhu and He, 2023). Gingerol (GN) and Shogaol (SG) are the major phenolic compounds in ginger, which exhibit multiple biological activities, such as antioxidant, anti-tumor, anti-bacterial, anti-inflammatory, immunomodulatory, and so on (Mao et al., 2019). Due to these properties, ginger can be used in food and medicine for disease prevention and treatment, attracting considerable interest from researchers. Based on current knowledge, plant cells can release large amounts of exosome-like nanoparticles whose structures and functions are similar to mammalian-derived exosomes (MDEs) (Logozzi et al., 2022; Kim et al., 2022; Zhu and He, 2023). These vesicles regulate plant physiological functions (e.g., innate immunity) and exhibit pharmacological activity (Ju et al., 2013; Dad et al., 2021). Mu and colleagues isolated and characterized the ginger-derived exosome-like nanoparticles (GELNs) from the obtained ginger juice, indicating that ginger contains exosome-like nanoparticles similar to those found in other plants, such as grapes, grapefruits, and carrots. Interestingly, compared to crude extracts of ginger, GELNs may offer a better therapeutic effect on gut-related inflammatory disease, as intestinal macrophages or monocytes can preferentially take them up through caveolin-dependent endocytosis (Mu et al., 2014; Jiang et al., 2022; Yin et al., 2022). In addition, Zhang and colleagues demonstrated that GELNs are rich in bioactive compounds from ginger, which possess anti-inflammatory and anti-cancer properties, such as 6-gingerol and 6-shogaol. Oral administration of GELNs can treat inflammatory bowel disease and colitis-associated cancer (CAC), representing a novel cell-free therapeutic approach (Zhang et al., 2016a). GELNs have garnered more attention and extensive research than other plant-derived exosome-like nanoparticles (PELNs) in the late decade. Researchers recognize that GELNs represent a novel, natural drug delivery system (DDS) for disease prevention and treatment. GELNs as drug carriers have advantages in biocompatibility and production yield compared to synthetic nanoparticles. Previous studies have demonstrated that researchers can use GELNs to deliver small molecular drugs and small RNAs for targeted therapy of intestinal tumors. Recently, Cui and colleagues took advantage of gastric acid tolerance of GELNs to deliver tetrahedral framework nucleic acids (tFNAs), targeting the modulation of intestinal flora to improve Parkinson’s disease (PD) (Cui et al., 2024). This study suggested that the GELN-based DDS can facilitate the strategy for treating brain diseases through the microbial-gut-brain (MGB) axis. This finding sparked our thinking that GELNs are worth further research to explore new candidates for drug carriers. The International Society for Extracellular Vesicles (ISEV) still does not have detailed naming rules for PELNs (Bai et al., 2024). Therefore, this review will use the term GELNs to introduce their characteristics and research progress in drug delivery to enrich our knowledge of this field. Finally, we will discuss the challenges and prospects of the GELN-based DDS.

## 2 Characteristics of GELNs as drug carriers

### 2.1 Physical characterisation

GELNs are nano-sized vesicles with sizes ranging from 100 to 500 nm. Under transmission electron microscopy (TEM) imaging, they exhibit a round, teacup-shaped morphology similar to that of MDEs. In contrast, atomic force microscopy (AFM) and scanning electron microscopy (SEM) images reveal that GELNs have a spherical shape and demonstrate good size homogeneity. GDENs also possess a lipid bilayer membrane structure similar to MEs and other PELNs. This membrane structure protects the content of GELNs from destruction and degradation by external actors, and even they are stable in stomach-like and intestine-like solutions (Mu et al., 2014; Di Raimo et al., 2023). In addition, this membrane structure facilitates the transport of GELNs across biological membranes by membrane-to-membrane fusion, theoretically allowing them to overcome barriers such as the blood-brain barrier (BBB) (Zhu and He, 2023). When exosomes encounter a polar medium, such as a hydrophilic buffer, they spontaneously acquire surface electrical charges because of electron affinity differences between the two phases, ionization of exosome membrane surface groups, and differential ion adsorption from electrolyte solutions (Beit-Yannai et al., 2018). The zeta potential can reflect the surface charges of the exosomes, and its value indicates charge stability. Previous research has shown that the zeta potential of GELNs typically ranges from −10 to −30 mV (Zhu and He, 2023). Zeta potential enables GELNs to easily combine with drugs, small molecules, or functional groups with positive charges through simple co-incubation.

### 2.2 Compositions

The four major components identified in GELNs include lipids, proteins, nucleic acids, and secondary metabolites, all of which play crucial roles in the function and stability of GELNs. GELNs contain a high proportion of phosphatidic acid (PA), which is involved in cytoskeletal rearrangements, vesicular transport, secretion, and endocytosis. PA facilitates the uptake of GELNs by recipient cells and participates in the preventive effect of GELN on obesity and insulin resistance and inhibitory effect on the growth of *P. gingivalis* (Zhang et al., 2016a; Sundaram et al., 2019; Kumar et al., 2022; Wei et al., 2023). GELNs contain specific active ingredients distinctive from ginger, such as gingerols and shogaols (Man et al., 2021). A previous study has shown that GELNs are rich in 6-gingerol, 8-gingerol, 10-gingerol, and 6-shogaol, and the levels of these active ingredients are much higher than those found in ginger slices. In addition, microRNAs (miRNAs) derived from GELNs can play a cross-kingdom physiological role in humans. Yin and colleagues identified 27 miRNAs with higher expression levels in GELNs that regulate human inflammatory and cancer-related pathways (Yin et al., 2022). Recent research has demonstrated that GELN-derived osa-miR164d can regulate reprogramming macrophage polarization, helping alleviate colitis-related symptoms (Yan et al., 2024). GELN-derived osa-miR-530-5p can inhibit the replication of severe acute respiratory syndrome coronavirus 2 (SARS-CoV-2) after GENPs accumulate in lung tissues (Kalarikkal and Sundaram, 2021). These findings suggested that GENPs can provide extra synergistic therapeutic effects in drug delivery. Therefore, researchers can use GELNs as drug carriers to encapsulate hydrophilic drugs within their aqueous core while accommodating hydrophobic drugs in their lipid bilayer.

### 2.3 Cellular uptake and biodistribution

Cellular internalization of PELN is crucial for drug delivery into recipient cells. This process involves multiple pathways, such as direct fusion, receptor-mediated fusion, clathrin-mediated endocytosis, raft-mediated endocytosis, caveolae-mediated endocytosis, phagocytosis, and macropinocytosis. Existing evidence indicates that GELNs can be internalized by intestinal Caco-2 Cells through caveolin-mediated endocytosis and macropinocytosis. Researchers proposed that the cellular uptake mechanism of GELNs in the small intestine may be related to the number of surface-binding proteins of the recipient cells or the characteristics of GELNs (Yin et al., 2022). In addition, some researchers have suggested that the lipid bilayer of GELNs could facilitate fusion with tumor cells (Zhang et al., 2016b). Zhuang and colleagues observed the specificity of endocytosis pathways in liver cells between the harvested GELNs from different sucrose gradients after sucrose gradient centrifugation. They isolated GELNs-1 from the 8/30% interfaces of the sucrose gradient and GELNs-2 from the 30/45% interfaces of the sucrose gradient, respectively. They found that GELNs-1 was internalized via macropinocytosis in primary hepatocytes, while GELNs-2 was internalized through microtubule-dependent active transport. Moreover, they found that external temperature will affect the cellular uptake of GELNs. Uptake rates of GELNs were very slow at 4°C but increased with the raised temperature, indicating that the uptake efficiency of GELNs depends on the temperature and metabolic energy (Zhuang et al., 2015). These findings suggested that various cell types can internalize GELN through different pathways, and this process is influenced by the surface-binding proteins of the recipient cells, the characteristics of the GELNs, and temperature. The specificity of various cells in the cellular uptake of GELNs will also impact the biodistribution of GELNs. However, research on the internalization pathways of GELNs in different cell types remains limited, and the mechanism behind the cellular uptake of GELNs in specific receptor cells is still not fully understood.

In addition to cellular uptake, the administration of GELN-based DDS will impact the biodistribution of GELNs. Existing literature reported the cases of oral and intravenous administration of GELNs in experimental animals (see Table 1). GELNs do not have the pungent taste associated with raw ginger. Therefore, oral administration is a safe and feasible route to GELN delivery and contributes to GELN accumulation inside the intestine for a long time (Xie et al., 2024; Zhao et al., 2024). However, small amounts of GELNs are also distributed in other organs, such as the liver, kidney, and spleen (Huang et al., 2024). Folic acid (FA) labeled on GELNs can enhance the GELN accumulation in the distal small intestine and colon rather than other organs, indicating that engineered GELNs with functional groups or targeting ligands have better biodistribution in the target tissue than native GELNs (Wang et al., 2019). Intravenous administration of GELNs avoids the first-pass effect of GELNs. GELNs are stable in the bloodstream for a long time, contributing to accumulating in tissues outside the gastrointestinal tract, such as tumor tissue. Intravenous administration is the priority one for GELN-based DDS in tumor-bearing animal models. In addition, the intranasal approach of GELNs would be a worthy attempt to improve their biodistribution in brain tissue, thereby expanding the application of GELN-based DDS in brain diseases.

**TABLE 1:** Literature examples of drug loading of GELNs.

Nomenclature of GELNs	Therapeutic agent	Engineering method	Engineering process	Loading capacity	Encapsulation efficiency	References
Ginger vesicles (GVs)	10-hydroxycamptothecin (HCPT)	Co-incubation	This mixture of HCPT and GV was incubated in water bath incubators at 37°C, and shaken for 12 h	21.32%	80.26%	Liu et al. (2025)
Ginger-derived exosome-like nanoparticles (GDNPs)	Indocyanine green (ICG)	Co-incubation	The mixture of ICG and GDNPs was stirred at 37°C for 3 h in the dark	19.86%	-	Guo et al. (2025)
Ginger derived exosome-like nanovesicles (GDENs)	Survivin siRNA	Co-incubation	GDENs and siRNAs were mixed in 100 μL of 1 × PBS with 2.5 μL of ExoFect Exosome transfection and incubated at 37°C for 1 h	80%	-	Li et al. (2018)
Ginger-derived exosome-like nanovesicles	Tetrahedral framework nucleic acids modified with antimicrobial peptides (Tac)	Electroporation	Tac and exosome-like nanovesicles were electrotransfected using Gene Pulser Xcell (Bio-Rad, CA, United States) at 20°C–25°C (0.7 KV, 350 Ls, pulsed 20 times). Then the mixture was incubated at 37°C for 30 min	76.18% ± 7.10%	-	Cui et al. (2024)
Ginger-derived nanovesicles (GDNVs)	Curcumin (CUR)	Sonication	CUR and GELNs were treated with ultrasound at 20% amplitude for 6 cycles (1 min per cycle and a 2 min interval between cycles) and then incubated at 37°C for 1 h to restore the structure	94.027% ± 0.094%	89.300% ± 0.344%	Mao et al. (2021)
GDNVs (Nanovectors of GELN lipids)	Doxorubicin (DOX)	Extrusion	Lipids and Dox were mixed to obtain a thin film. Then the film was passed through an extruder (AVESTIN) with a 200 nm polycarbonate membrane 20 times	95.9% ± 0.26%	-	Zhang et al. (2016b)

## 3 Loading drug methods of GELN-based drug delivery system

At present, the engineering methods for loading drugs into exosomes can be simply classified into donor cell and exosome engineering (Jiang et al., 2022). Donor cell engineering requires drugs or therapeutic agents to be taken up by donor cells and then encapsulated into exosomes through the cargo-sorting mechanism during exosome biogenesis. Plant cells can produce PELNs under biotic and abiotic stresses, such as pathogen infections. PELN biogenesis involves the multivesicular bodies (MVBs) pathway, which starts from the inward budding of the cytoplasmic membrane to form endocytic vesicles, internalizing various components (e.g., lipids, proteins, small molecules, and diverse metabolites) to form early endosomes. Then, early endosomes mature to form late endosomes, known as MVBs, which either fuse with lysosomes, resulting in degradation, or dock the cytoplasmic membrane to release PELNs (Lian et al., 2022; Zhao et al., 2024). This pathway also requires the action of the endosomal sorting complex required for transport (ESCRT) complexes (e.g., ESCRT-0, I, II, and III) for cargo sorting (Kim et al., 2022). In addition, there are two other pathways in PELN biogenesis: the vacuolar pathway and the exocyst-positive organelle (EXPO) pathway. The former involves the small vacuoles (SVs) derived from MVBs fusing with the central vacuole, then the central vacuole releases vacuoles, which dock the cytoplasmic membrane to release PELNs. The latter, an unconventional secretion route, involves the double-membrane EXPO fusing with the plasma membrane to release PELNs (Bai et al., 2024; Zhao et al., 2024). Although PELNs can theoretically encapsulate drugs using the donor cell engineering method similar to MDEs, few studies have used the donor cell engineering method. Instead, in contrast, some exosome engineering methods, such as co-incubation, electroporation, sonication, and extrusion, have been utilized for drug loading of GELNs (see Table 1). a) Co-incubation: GELNs can load hydrophobic drugs into the hydrophilic core and positively charged drugs on their lipid membrane by electrostatic interactions following simply mixing and stirring. This method is simple and easy to operate. However, its drug-loading efficiency may be susceptible to the hydrophobicity and charges of drugs, and the entrapment efficiency of hydrophilic drugs may be unsatisfactory. b) Electroporation: This method requires GELNs mixed with drugs in the electroporation buffer, and then an electroporator generates the electric field to disturb the lipid bilayer of GELNs, creating some transient tiny pores that allow drugs to penetrate the GELNs by a concentration gradient. The pores are closed to avoid drug leakage after removing the electric field. c) Sonication: This method requires the mechanical shear force of ultrasound to compromise the membrane integrity of GELNs, which facilitates drugs to penetrate GELNs by a concentration gradient. After the ultrasound intervention ends, the membrane integrity will automatically recover to avoid drug leakage. d) Extrusion: This method needs a lipid extruder to generate a mechanical extrusion load that pushes GELNs or their lipid film along with drugs across a porous membrane multiple times, resulting in the generation of uniform-sized drug-loaded new nanosized vesicles. However, the repeated and intense extrusion may affect membrane potential and protein structures, leading to characteristics of the new nanosized vesicles that differ from those of the native GELNs. The existing experimental evidence indicates that sonication and extrusion provided a high loading capacity in hydrophobic drugs, and electroporation may be suitable for nucleic acid loading, which provides a reference for future research on GELN-based DDS.

## 4 Applications of GELN-based drug delivery system

Researchers have suggested that GELNs serve as effective drug carriers due to their good drug-loading capacity and ability to penetrate the small intestine. In particular, the absorption trend of GELNs in the duodenum is better than in the jejunum and ileum (Man et al., 2021). As a result, many current studies utilize oral GELN-based DDS to treat intestinal and extraintestinal diseases by regulating intestinal function and gut microbiota, and intravenous administration was suitable for anti-tumor treatment (see Table 2).

**TABLE 2:** Literature examples of GELN-based DDS in disease treatment.

Disease	Therapeutic agent	Name of DDS	Engineering method	Animal model	Route of administration	Drug dosage	Observation period	References
Inflammatory bowel disease (IBD)	Infliximab (INF) loaded LMSN	INF/LMSN@GE	Extrusion	Colitis mice	Oral administration	10 mg/kg (INF), each day	16 days	Mao et al. (2021)
	Curcumin	CG	Sonication	Colitis mice	Oral administration	10.63 mg/mL (GDNVs)	7 days	Huang et al. (2024)
Colon cancer	Doxorubicin (DOX)	Dox-FA-GDNVs	Extrusion	Tumor-bearing mice	Intravenous administration	100 μg/dose (DOX), every 4 days	20 days	Zhang et al. (2016b)
Breast cancer	10-hydroxycamptothecin (HCPT)	GV-HCPT	Co-incubation	Tumor-bearing mice	Intravenous administration	200 μg/mL (HCPT)	14 days	Liu et al. (2025)
Breast cancer	Indocyanine green (ICG)	GDNPs@ICG	Co-incubation	Co-incubation	Intravenous administration	3.75 mg/kg (ICG), 3 times a week	14 days	Guo et al. (2025)
Oral cancer	Survivin siRNA	FA-3WJ/GDENs/siSurvivin	Co-incubation	Tumor-bearing mice	Retro-orbital injection	0.1 pmole GDENs/0.5 nmole RNA per mice, every 2 days	14 days	Li et al. (2018)
Parkinson’s disease (PD)	Tetrahedral framework nucleic acids (tFNA) modified with antimicrobial peptides (AMP)	Exo@tac	Electroporation	PD mice	Oral administration	200 μL (Exo@tac), each time, 3 times a day	60 days	Cui et al. (2024)
Hereditary hemochromatosis (HH)	Divalent metal transporter 1 siRNA	FA-GDLVs/siRNA	Extrusion	HH mice	Oral administration	3.75 nmol GDLVs/siRNA per dose, each day	16 days	Wang et al. (2019)
Type 2 diabetes mellitus (T2DM)	Ammonia borane loaded HMS	HMS/A@GE	Sonication	T2M mice	Oral administration	5 mg/kg (HMS@GE), every 2 days	54 days	Wang et al. (2025)

### 4.1 Intestinal disease

Inflammatory bowel disease (IBD), including Crohn’s disease and ulcerative colitis (UC), is a nonspecific chronic inflammatory disorder of the gastrointestinal tract characterized by complex, relapsing, and refractory symptoms (Seyedian et al., 2019). TNF-α is one of the key pro-inflammatory cytokines involved in the pathogenesis of IBD, making it a critical target for treatment (Souza et al., 2023). The anti-TNF-α antibodies include infliximab (INF), golimumab, certolizumab pegol, and adalimumab. However, whether subcutaneous, intramuscular, or intravenous administration of them may cause serious adverse effects. Mao and colleagues used GELNs to encapsulate INF-containing mesoporous silicon nanoparticles (LMSN) using an extrusion method based on a Micro-extruder with 400 nm porous membranes (Avanti). They named this developed GELN-based drug delivery system INF/LMSN@GE. They treated the colitis mice with oral administration of INF/LMSN@GE, demonstrating that INF/LMSN@GE showed good stability in the gastrointestinal tract, colon-targeted delivery ability, and high intestinal epithelium permeability due to the exit of GELNs. Once INF/LMSN@GE were taken up by intestinal epithelial cells through membrane fusion or endocytosis, INF/LMSN was released and crossed the intestinal epithelial cells into the lamina propria, where TNF-α is concentrated. Meanwhile, GELNs provided additional anti-inflammatory effects, allowing INF/LMSN@GE to show a significantly greater efficacy, which is better than intravenously administered INF. Therefore, they suggested that GELNs could serve as a new oral DDS for colon-targeted delivery of anti-TNF-α antibodies, avoiding systemic immunosuppression (Mao et al., 2021).

Recently, Huang and colleagues used GELNs to carry curcumin (CUR) to overcome the limitations of CUR in UC treatment, such as physiological instability and low concentration at the intestinal lesions. In their study, GELNs can load CUR at a high loading capacity and encapsulation efficiency after sonication. They found that CUR-loaded GELNs (CG) can be primarily distributed in the colon of UC mice after oral administration and showed better anti-UC activity than free CUR and GDNV. The therapeutic mechanism of CG is possibly related to its anti-inflammation, anti-oxidative stress, modulation of intestinal barrier function, and anxiety relief effects (Huang et al., 2024).

### 4.2 Cancer

Doxorubicin (Dox) is one of the most commonly used chemotherapy drugs for cancer treatment, such as colon cancer. However, the clinical therapeutic outcomes of Dox in treating colon cancer are often unsatisfactory due to its off-target toxicity and prominent hydrophobicity (Zhao et al., 2023a). Exploring the effective delivery of DOX by nanotechnology and biomaterials is meaningful for colon cancer treatment. Zhang and colleagues designed ginger-derived nanovectors (GDNVs) based on the lipids of GELNs to serve as the delivery platform for Dox in colon cancer treatment. They first harvested the GELNs from ginger juice and then extracted the total lipids of GELNs using the Bligh and Dyer method. They mixed lipids, Dox, and folate (FA) in chloroform and dried the mixture under nitrogen to obtain a thin lipid-complex film. This film was passed through a liposome extruder with a 200 nm polycarbonate membrane to form FA and Dox-containing GDNVs (Dox-FA-GDNVs). They observed that the negatively charged lipid components contribute to GDNVs effectively encapsulating slightly positively charged Dox by electrostatic interaction. The presence of FA enhances tumor targeting of GDNVs. The Dox-FA-GDNVs exhibited a longer circulation time in the bloodstream, greater antitumor efficacy, and lower systemic toxicity than free Dox in tumor-bearing mice. In addition, Dox-GDNVs could release loaded Dox more rapidly in acidic pH close to the tumor microenvironment than commercially available liposomes (Zhang et al., 2016b). Recently, Liu and colleagues reported that GDNVs have a good drug loading efficiency after being incubated with positively charged 10-hydroxy-camptothecin (HCPT) for 12 h at 37°C in the presence of 0.1 M Ca2+. HCPT-loaded GDNVs (GV-HCPT) displayed remarkable inhibitory effects on tumor growth in 4T1-bearing mice, exhibiting excellent target delivery ability and good biocompatibility. Notably, the bioactive compounds in GDNVs, such as gingerol, ginger phenol, and other active substances, can synergistically enhance the inhibitory effects on tumor cells when combined with HCPT. These findings suggest that GDNV-based DDS provides a reliable approach for chemotherapy (Liu et al., 2025).

Survivin (also known as BIRC5), a cancer-associated protein, is highly expressed in different types of cancers. It plays a significant role in tumor proliferation, recurrence, and chemotherapy resistance, making it an attractive target for cancer treatment (Fang et al., 2024; Wijaya et al., 2024). The gene silencing by survivin siRNA (siSurvivin) has attracted more interest in cancer treatment. Li and colleagues were the first to explore the role of GELNs in the systemic delivery of siSurvivin. They fused a survivin siRNA into FA-labeled packaging RNA three-way junction (pRNA-3WJ), while cholesterol-conjugated pRNA-3WJ interacted with the GELN membrane to form FA-3WJ/GDENs/siSurvivin complex. After intravenous administration in xenograft mouse models, this complex effectively targeted KB cells and reduced survivin expression, leading to cancer suppression. This finding suggested that FA ligands can enhance the tumor-targeting ability of GELNs, improving the uptake of GDENs by cancer cells, and GELNs can serve as the carriers for systemic delivery of siRNA in cancer treatment after being engineered by ligand-displaying arrow-tail RNA nanoparticles (Li et al., 2018).

Indocyanine green (ICG) is a photosensitizer for photodynamic therapy (PDT) and can produce reactive oxygen species (ROS) and heat to kill cancer cells when exposed to NIR light (Mahmut et al., 2023). Several disadvantages of ICG, such as poor stability in aqueous solutions, the tendency to easily aggregate, and a short plasma half-life, limit its wider application in cancer treatment. Guo and colleagues utilized GENPs as the carriers to load ICG by co-incubation to improve the bioavailability and tumor targeting of ICG. They observed that both GENPs and GDNPs@ICG can be taken up and effectively internalized by 4T1 cells via a lipid-dependent pathway and then accumulated in the endoplasmic reticulum (ER), mitochondria, and lysosomes. In animal experiments, the 4T1-tumor-bearing mouse models received ICG-loaded GENPs (GDNPs@ICG) by intravenous injection and then irradiated with an 808 nm laser. They found that GENPs did not affect the photothermal performance and ROS production of ICG *in vivo*, leading to photodynamic and photothermal effects in tumor tissue. In addition, GENPs can release lipids, 6-shogaol, and other components, enhancing the anti-breast tumor therapy effects by inducing lipid peroxidation and enhanced ER stress. They suggested that the combination of GENPs and ICG can effectively kill tumor cells through multiple mechanisms, including photothermal ablation, ROS-induced oxidative damage, lipid peroxidation, ER stress, reduced angiogenesis, inhibited metastasis, activated anti-tumor immune escape, and promotion of tumor cell senescence, indicating that GENPs can be an optional DDS in anti-tumor PDT (Guo et al., 2025).

### 4.3 Neurodegenerative diseases

PD is a common neurodegenerative disease, and its occurrence and development are closely associated with the microbiota-gut-brain axis (MGBA) (Lei et al., 2021). Cui and colleagues chose gut microbiota and MGBA as the therapeutic targets and used tFNAs to modulate gut microbiota. In their study, GELNs encapsulated the antimicrobial peptides (AMP)-modified tFNAs by electroporation to form Exo@tac. They found that oral administration of Exo@tac improved motor symptoms and pathological characteristics associated with PD in mouse models. Exo@tac can regulate PD-related intestinal bacteria and affect MGBA-related macrophages, neuromicroglia, and intestinal endocrine cells, reducing inflammation and apoptosis in the central nervous system, promoting the production of neurotransmitters 5-HT and the dopamine precursor, as well as reducing deposition of the PD signature protein alpha-synuclein (a-syn). Notably, GELNs can stay in the gastrointestinal tract for a long time without being toxic to vital organs, protecting tFNAs from gastric acid and even improving the damage in the stomach and intestine. These findings suggested that this kind of GELN-based DDS represents a novel strategy for PD drug development and the innovative delivery of nucleic acid nanomedicines (Cui et al., 2024).

### 4.4 Iron overload disorders

Hereditary hemochromatosis (HH) is an autosomal recessive genetic disease characterized by an excessively excessive absorption of dietary iron without an effective excretory mechanism, leading to iron overload and subsequent organ damage (Fleming and Sly, 2002; Katsarou et al., 2019). Divalent metal transporter 1 (DMT1) is the major iron transporter and contributes to ferrous iron uptake in enterocytes (Yanatori and Kishi, 2019). Wang and colleagues suggested that intestinal Dmt1 is essential for iron loading in murine models of HH (Hepc−/− mice), and targeting intestinal Dmt1 may attenuate iron loading. They developed FA-conjugated GDLVs carrying Dmt1 siRNA (FA-GDLVs/siRNA) following the method introduced by Zhang et al. They mixed lipids, Dox, and FA to obtain a thin lipid-complex film, then extruded it with a liposome extruder to form FA-GDLVs/siRNA. Depending on the targeting of FA and the protection effect from GDLVs, oral FA-GDLVs/siRNA can effectively deliver Dmt1 siRNA to epithelial cells of the duodenum and proximal jejunum with high DMT1 expression rather than portal blood circulation and lymph system or degradation by RNase. Dmt1 mRNA expression and serum ferritin levels remarkably decreased in Hepc−/− mice treated with FA-GDLVs/siRNA, demonstrating that GDLVs are reliable carriers of functional Dmt1 siRNA, providing a novel treatment modality for HH (Wang et al., 2019). In the subsequent research, they also found that GDLVs reduced iron absorption in intestine by unidentified bioactive components, contributing to the positive outcomes of HH treatment (Wang et al., 2021).

### 4.5 Diabetes mellitus

Diabetes mellitus (DM) is a chronic metabolic disease that can lead to serious complications, such as retinopathy, nephropathy, and peripheral neuropathy, affecting the health and quality of life of patients (Bailes, 2002). It is well known that insulin resistance and pancreatic β-cell dysfunction are the primary pathogenesis of type 2 DM (T2DM), representing the key issues in treating T2DM. The roles of gut microbiota and microbial metabolites in the pathogenesis and therapy of T2DM have attracted widespread attention (Zhou et al., 2022; Crudele et al., 2023; Wu et al., 2023). Lately, Wang and colleagues reported a non-drug strategy for T2DM integrating the antioxidant and anti-inflammatory effects of hydrogen (H_2_) therapy and gut microecological modulation of GELNs. They first used amino-modified hollow mesoporous silica (HMS-NH_2_) nanoparticles to encapsulate H_2_ donor ammonia borane (A) to form HMS/A. Then, HMS/A was covered with GELNs to form HMS/A@GE using sonication from an ultrasonic cell disruptor. They found that the blood glucose levels of T2M mice significantly declined at the end of the experiment after orally administering HMS/A@GE once every 2 days. This antidiabetic efficacy is attributed to the H_2_ therapy and GELNs. The former suppresses oxidative stress in the liver and pancreas and the inflammatory response, protecting islet β-cell function and improving insulin resistance. The latter cooperates with H_2_ therapy to remodel the composition and metabolites of the gut flora, maintaining intestinal mucosal-barrier integrity and alleviating glucose dysmetabolism and liver steatosis. These findings suggested that HMS/A@GE provides a viable option for simultaneously solving insulin resistance and pancreatic β-cell dysfunction (Wang et al., 2025).

## 5 Challenges of GELN-based drug delivery system

Although the above research progress has achieved encouraging results, there are still some critical challenges that limit the rapid development of GELN-based DDS in standardized production and clinical applications.

### 5.1 Nomenclature

The current nomenclature of GELNs also includes ginger-derived nanoparticles (Zhuang et al., 2015), ginger-derived exosome-like nanovesicles (Li et al., 2018), ginger-derived edible nanoparticles (Kalarikkal et al., 2020), ginger-derived exosomes (Mao et al., 2021), ginger-derived extracellular vesicles (Hao et al., 2024), and ginger vesicles (Liu et al., 2025). The lack of standardization in nomenclature will lead to misunderstandings regarding the biogenesis pathways and functions of native and engineered GELNs. For instance, the engineered GELNs produced by the extrusion method are new artificial vesicles that may have characteristics differing from those of native GELNs. The International Society for Extracellular Vesicles (ISEV) has published the latest version of the expert consensus on the studies of extracellular vesicles, Minimal information for studies of extracellular vesicles (MISEV2023). MISEV2023 defined the EV nomenclature and related terms, including extracellular vesicles (EVs), non-vesicular extracellular particles, EV mimetics (EVMs), artificial cell-derived vesicles (ACDVs), exosomes, exosome-like vesicles, and so on. MISEV2023 discouraged using “Exosome-like vesicles” or similar terms to describe EV-like particles produced through methods such as direct disruption of cells, *de novo* synthesis from molecular components, and fusion of native EVs with, for example, liposomes. Instead, it recommended the use of EV mimetics. In addition, MISEV2023 recommended using ACDVs to define the EV mimetics produced by extrusion in the laboratory under certain conditions. MISEV2023 suggested that its defined nomenclature can apply to all EV studies. However, the source of EV-containing materials described did not mention plant cells (Welsh et al., 2024). Recently, the Chinese Expert Committee on Research and Application of Chinese Herbal Vesicles published the Consensus statement on research and application of Chinese herbal medicine-derived extracellular vesicles-like particles (2023 edition), aiming to provide a reference in the research and application of Chinese herbal vesicles. This consensus statement recommended using the English designation from the most recent edition of the Chinese Pharmacopoeia for nomenclature of specific Chinese herbal medicine. For instance, GELNs can be named Zingiber officinale Roscoe-derived EV-like particles. However, there remains a lack of consensus on the nomenclature for engineered Chinese herbal vesicles (Zhao et al., 2023b). Given that the nanovesicles from GDENs or GELN lipids by the extrusion method represent an artificial method to create “Ginger-derived nanovesicles” rather than a natural biogenesis process. These nanovesicles are suitable for naming as artificial ginger-derived vesicles. Researchers also have to describe the extraction methods of natural GELNs to reduce the nomenclature misunderstandings for readers.

### 5.2 Safety

The safety of DDS is a crucial challenge for its clinical translation and application. Thus far, many researchers believe that PELNs have better safety profiles because they do not harbor zoonotic or human pathogens. Previous experimental research indicated that intravenous injection and oral administration of GELNs have shown no evidence of potential toxicity in hemolysis, liver injury indices (aspartate aminotransferase and alanine aminotransferase), kidney injury indices (creatinine and blood urea nitrogen), and the histological analysis of major organs (heart, liver, spleen, lung, and kidney) of experimental animals. However, the existing safety data of experimental animals is not yet sufficient to address the concerns of researchers. The critical analysis of dose-dependent toxicity, long-term safety, and potential immunogenicity is still absent. Moreover, engineering the surface of GELNs with functional groups or targeting ligands can endow the extra function expected by researchers in the transportation of drugs, making we can not ignore the unexpected change in GELNs brought about by engineering methods and their application of relevant chemical reagents, which could potentially lead to side effects, including general toxicity, immunogenicity, immunotoxicity, and tumorigenicity. A completed clinical trial (NCT04879810) has investigated the GELNs with and without curcumin in IBD treatment, and we will know the safety and tolerability of GELNs in patients once the results are published.

### 5.3 Heterogeneity

The secretion of GELNs is a response mechanism for ginger cells to cope with environmental stresses. Factors such as temperature, humidity, fertilizers, and insect pests can influence the biogenesis and biological functions of GELNs. In addition, various elements, including the species, production area, land conditions, harvest time, and age of ginger, can affect the biochemical characteristics of GELNs, including lipids, proteins, miRNAs, and active ingredients. Unlike MDEs harvested from cell culture media, body fluids, or tissues, GELNs are from ginger juice obtained by cutting and crushing ginger at high speeds using a wall breaker or blender. Researchers typically filter or centrifuge the crude ginger juice to remove large fibers and cell debris before applying differential centrifugation (DC) to harvest GELNs. The harvested GELNs may undergo further purification through sucrose gradient ultracentrifugation. DC plus sucrose density gradient ultracentrifugation is the most classical and commonly used method (Yin et al., 2022). Indeed, researchers will choose the extraction and purification methods depending on their purpose, experimental equipment, and cost. The available technologies include ultracentrifugation, density gradient centrifugation, ultrafiltration, commercial exosome isolation kits, etc. Some researchers have introduced and compared the advantages and disadvantages of these available methods in their published reviews (Lian et al., 2022; Zhu and He, 2023; Zhao et al., 2024). In practice, Huang and colleagues suggested that ultracentrifugation and density gradient centrifugation can provide high purity of GELNs. Still, the disadvantages of this method are the long centrifugation time, high centrifugation equipment requirements, and low extraction rate. PEG8000 can easily obtain GELNs without prolonged high-speed centrifugation by allowing exosomes to aggregate and settle, but the purity of GELNs is low. Integration of these methods can improve the purity and yield of GELNs and reduce instrument losses (Huang et al., 2024). The yields of GELNs by different extraction methods and their effects on the size and zeta potential of GELNs were described in detail in the published review from Zhu and colleagues. We can learn that varying methods lead to different yields of GELNs, and even using the same method can produce inconsistent results across different studies. Current pretreatment, separation, and purification technologies inevitably result in losses of GELNs, causing variability between different batches of GELNs and complicating large-scale production. Of course, the changes in the size and zeta potential of GELNs may affect the drug-loading efficiency, targeting ability, distribution, and metabolism of engineered GELNs using different engineering methods, ultimately affecting therapeutic efficacy.

### 5.4 Storage

The stability of GELNs in stomach-like and intestine-like solutions, along with their prolonged circulation time in the bloodstream, makes them advantageous as drug carriers. However, the morphology, size, and zeta potential of GELNs are easily affected by the pH value of the external environment (Suresh et al., 2021). Other external factors, such as temperature, illumination, and oxygen, can inactivate the active ingredients in GELNs. Proper storage of GELNs and engineered GELNs is crucial to clinical translational research. GELNs can be stable at 4°C for 25 days, and retain biological functions like the freshly extracted GELNs after being stored at −80°C. Some researchers suggested that cellular cryoprotectants or stabilizers may be suitable for GELN storage (Zhu and He, 2023). However, only a few studies mentioned storing harvested GELNs or engineered GELNs at −20°C for later use. Thus far, there is no unified standard protocol for the storage parameters of GELNs, including recommended temperature, quality guarantee period, freeze-thaw times, and preservation solution. The exploration of the storage parameters of GELN is still far from sufficient.

## 6 Summary and outlook

In recent years, the research on PELNs has attracted more and more attention due to their potential as a new class of cell-free therapeutic approaches and natural DDS. Ginger has a long history as a cooking seasoning and herbal remedy. Oral ginger is non-toxic and offers various therapeutic benefits. GELNs possess pharmacological activity attributed to unique active ingredients from ginger, meanwhile, they are reliable carriers for delivering hydrophilic or hydrophobic drugs and small RNAs. Although this review has introduced the characteristics, research progress, and challenges of GELNs in drug delivery, the following points need attention and discussion.

**FIGURE 1:**
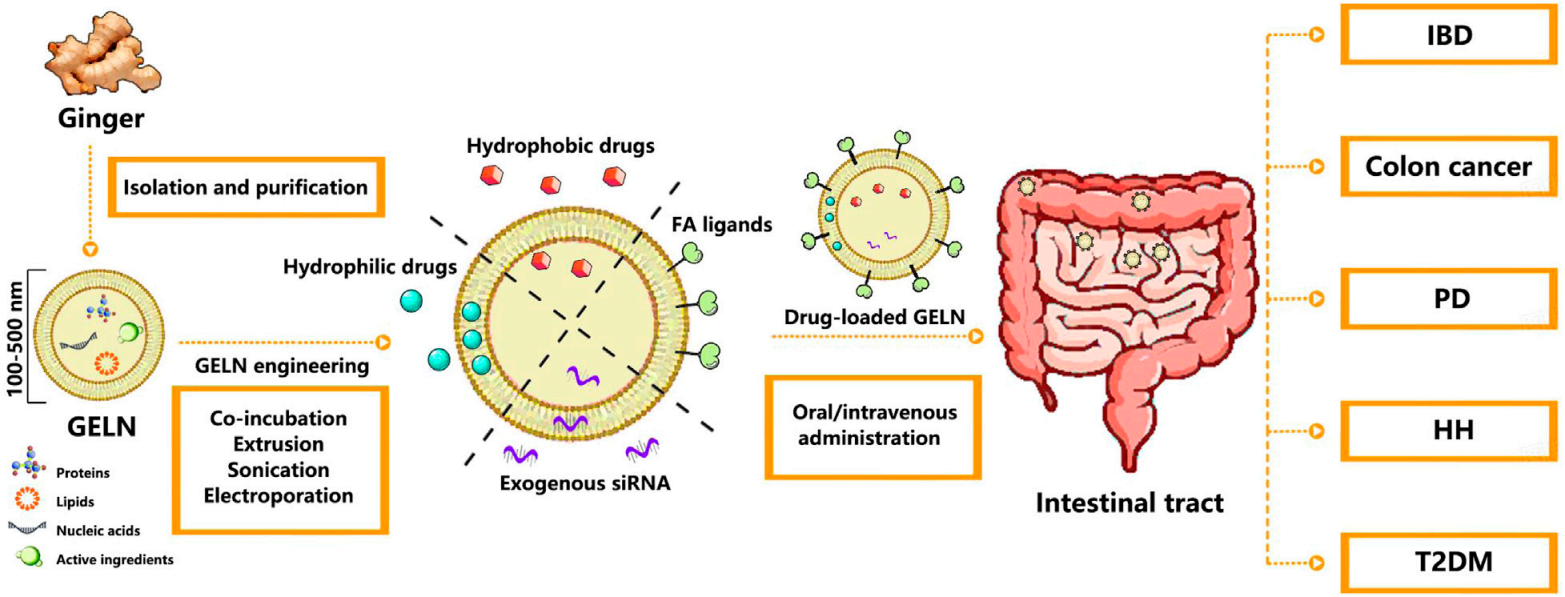
Schematic illustration of the preparation and application of drug-loaded GELNs in various disease treatments. Abbreviation: GELN, Ginger-derived exosome-like nanoparticle; FA, Folate; IBD, Inflammatory bowel disease; PD, Parkinson’s disease; HH, Hereditary hemochromatosis; T2DM, Type 2 diabetes mellitus.

First, many researchers harnessed the intestinal targeting ability of GELNs to efficiently deliver drugs to the intestine for intestinal inflammation, cancer, and extraintestinal disease treatment (see Figure 1). Intravenous administration of GELNs extends their therapeutic application beyond the gastrointestinal tract. Theoretically, GELNs may have the potential to cross the BBB like other PELNs (e.g., *Momordica charantia*-derived exosome-like nanoparticles and ginseng-derived exosome-like nanoparticles), making them suitable for delivering the drugs that cannot cross the BBB for brain disease treatment (Cai et al., 2022; Kim et al., 2023), and nasal administration of GELNs would be a good attempt compared to intravenous administration. Transdermal administration may help explore the effect of GELNs on conditions such as alopecia (Hao et al., 2024). GELNs can be suitable for various disease treatments by different routes of administration, providing the chance for them to serve as promising drug carriers.

Second, the lipid bilayer membrane structure of GELNs offers opportunities for coating drug-loaded nanomaterials, such as MSN, metal-organic framework (MOF), polymer nanomaterials, and nanotubes. GELNs can improve the biocompatibility of these nanomaterials, decrease their immunogenicity, and prevent drug leakage during blood circulation. GELNs can also encapsulate contrast agents, photosensitizers, photothermal agents, and H_2_ donors, facilitating applications in imaging diagnosis, PDT, sonodynamic therapy, and gas therapy. This versatility makes GELN-based DDS suitable for a wide range of biomedical applications. Moreover, GELNs can be directly conjugated with targeting ligands (e.g., aptamers, antibodies, and peptides) through click chemistry or lipid insertion, enhancing their targeting ability and drug delivery efficiency, similar to other engineered nanoparticles featuring lipid bilayer membrane structures. These properties of GELNs will also enrich the design of GELN-based DDS to help them become multifunctional carriers.

Third, GELNs represent a promising drug carrier, but exciting results come from animal experiments, and the research on GELNs remains in its early stages. Thus far, researchers have mainly focused on the biological function of GELNs and their potential benefits for disease treatment, and our understanding of them is limited, particularly regarding their biogenesis, cargo-sorting mechanism, pharmacological activity, absorption, distribution, excretion, and metabolism in the human body. Of course, some challenges still limit the GELN-based DDS from meeting the requirements of standardized production and clinical applications. These issues will also prompt researchers to explore them in-depth, and more basic and clinical research will be conducted. At present, an established clinical trial of CUR-loaded GELNs for treating IBD (NCT04879810) marks a promising beginning. In the future, more researchers will participate in the research and discussion of GELNs, from their biogenesis, cargo-sorting mechanism, optimization of engineering protocols, and standardization of large-scale production, to experimental or clinical trial design, and assist researchers in planning subsequent work.

Fourth, GELNs have shown similar yields and intestinal absorption trends compared to other PELNs derived from grapes, grapefruit, and carrots. However, the biological effects of GELNs differ from other PELNs on the recipient mammalian cells. For example, GELNs affect Nrf2 activation or induction of anti-inflammatory molecules, suggesting that ingesting them may play a role in maintaining intestinal homeostasis in terms of the production of pro- and anti-inflammatory cytokines (Mu et al., 2014). GELNs are well-suited for treating intestinal diseases. Several reviews have summarized the identification, characterization, biological functions, effects on human systemic diseases, and therapeutic potentials of GELNs and other PELNs (Kim et al., 2022; Mu et al., 2023; Yi et al., 2023; Bai et al., 2024). The anti-inflammatory, antioxidant, and anti-tumor properties of GELNs will provide potential benefits in extraintestinal disease treatments like other PELNs. Some literature examples mentioned in this review indicate that GELNs, as drug carriers, also provide potential benefits or enhance therapeutic efficacy. Therefore, the composition and biological functions of GELNs also need to be considered when designing GELN-based DDS.

Despite current challenges requiring more time and effort to overcome, the potential benefits of GELNs should not be ignored. Advancing the understanding of GELNs and the continuous progress of scientific research technology will promote the development of GELN-based DDS. Hence, we encourage future research in this direction to uncover new findings.

## Glossary

GN: Gingerol
SG: Shogaol
MDEs: Mammalian-Derived Exosomes
GELNs: Ginger-Derived Exosome-Like Nanoparticles
CAC: Colitis-Associated Cancer
PELNs: Plant-Derived Exosome-Like Nanoparticles
DDS: Drug Delivery System
tFNAs: tetrahedral Framework Nucleic Acids
PD: Parkinson’s disease
ISEV: International Society for Extracellular Vesicles
TEM: Transmission Electron Microscopy
AFM: Atomic Force Microscopy
SEM: Scanning Electron Microscopy
BBB: Blood-Brain Barrier
PA: Phosphatidic Acid
SARS-CoV-2: Severe Acute Respiratory Syndrome Coronavirus 2
FA: Folic Acid
MVBs: Multivesicular Bodies
ESCRT: Endosomal Sorting Complex Required For Transport
EXPO: Exocyst-Positive Organelle
SVs: Small Vacuoles
IBD: Inflammatory Bowel Disease
UC: Ulcerative Colitis
INF: Infliximab
CUR: Curcumin
Dox: Doxorubicin
GDNVs: Ginger-Derived Nanovectors
HCPT: 10-Hydroxy-Camptothecin
ICG: Indocyanine Green
ROS: Reactive Oxygen Species
ER: Endoplasmic Reticulum
PDT: Photodynamic Therapy
MGBA: Microbiota-Gut-Brain Axis
HH: Hereditary Hemochromatosis
DMT1: Divalent Metal Transporter 1
DM: Diabetes Mellitus
EVs: Extracellular Vesicles
EVMs: Extracellular Vesicle Mimetics
ACDVs: Artificial Cell-Derived Vesicles
DC: Differential Centrifugation
